# Human airway smooth muscle cell proliferation from asthmatics is negatively regulated by semaphorin3A

**DOI:** 10.18632/oncotarget.12884

**Published:** 2016-10-25

**Authors:** Hesam Movassagh, Nazanin Tatari, Lianyu Shan, Latifa Koussih, Duaa Alsubait, Mahdi Khattabi, Naresh S. Redhu, Michael Roth, Michael Tamm, Jamila Chakir, Abdelilah S. Gounni

**Affiliations:** ^1^ Department of Immunology, Rady Faculty of Health Sciences, Max Rady College of Medicine, University of Manitoba, Winnipeg, Manitoba, Canada; ^2^ Université de Saint-Boniface, Winnipeg, Manitoba, Canada; ^3^ Division of Gastroenterology, Hepatology and Nutrition, Boston Children's Hospital, Boston, MA, USA; ^4^ Department of Biomedicine, Pneumology & Pulmonary Cell Research, University Hospital Basel and University of Basel, Basel, Switzerland; ^5^ Centre de Recherche de l'Institut Universitaire de Cardiologie et de Pneumologie du Quebec, Universite' Laval, Quebec City, Canada

**Keywords:** airway remodeling, asthma, neuropilin 1, platelet-derived growth factor, semaphorin 3A, Pathology Section

## Abstract

Airway smooth muscle (ASM) hyperplasia is a key feature of airway remodeling in development of lung diseases such as asthma. Anomalous proliferation of ASM cells directly contributes to ASM hyperplasia. However, the molecular mechanisms controlling ASM cell proliferation are not completely understood. Semaphorins are versatile regulators of various cellular processes including cell growth and proliferation. The role of semaphorins in ASM cell proliferation has remained to be addressed. Here, we report that semaphorin 3A (Sema3A) receptor, neuropilin 1 (Nrp1), is expressed on human ASM cells (HASMC) isolated from healthy and asthmatic donors and treatment of these cells with exogenous Sema3A inhibits growth factor-induced proliferation. Sema3A inhibitory effect on HASMC proliferation is associated with decreased tyrosine phosphorylation of PDGFR, downregulation of Rac1 activation, STAT3 and GSK-3β phosphorylation. Bronchial sections from severe asthmatics displayed immunoreactivity of Nrp1, suggestive of functional contribution of Sema3A-Nrp1 axis in airway remodeling. Together, our data suggest Sema3A-Nrp1 signaling as a novel regulatory pathway of ASM hyperplasia.

## INTRODUCTION

Asthma is a chronic inflammation of conducting airways in association with airway hyperresponsiveness (AHR) and remodeling [1]. Structural changes in the airway walls, induced by a vicious circle of injury and repair processes, are collectively called airway remodeling. Increased airway smooth muscle (ASM) mass is a hallmark of airway remodeling which causes airway narrowing in asthma and chronic obstructive pulmonary disease [2].

Proliferation of ASM cells induced by a wide spectrum of mitogens (e.g. growth factors, cytokines, inflammatory mediators and allergens) has been proposed as a primary mechanism underlying increase ASM mass [3]. Several signaling pathways could be activated in parallel or as a cascade upon stimulation of ASM cells with mitogens [4]. It has been previously shown that Ras-related C3 botulinum toxin substrate 1 (Rac1) GTPase [5], signal transducer and activator of transcription 3 (STAT3) [5] and glycogen synthase kinase 3 beta (GSK-3β) [6] are actively involved in induction of ASM cell proliferation. Previous studies have addressed that inhibition of these pathways using exogenous specific inhibitors or gene silencing strategies reduces mitogen-induced ASM cell proliferation and could be considered as novel treatment options to minimize ASM hyperplasia. However, little is known about intrinsic mechanisms controlling these pathways which get dysregulated in ASM cells at pathological conditions.

Semaphorins, identified as a family of conserved secreted or membrane-bound axon guidance proteins, are involved in orchestration of various cellular processes including cell proliferation beyond the nervous system [7, 8]. It has been shown that semaphorin (Sema) family members Sema4A and Sema4D play a role in airway inflammation [9-12]; whereas Sema3E is involved in migration and proliferation of ASM cell [13].

Semaphorin 3A (Sema3A) can function as a chemorepulsive cue inhibiting axonal outgrowth during neural development [14]. Sema3A is also a putative tumor suppressor in breast, prostate and lung cancers [15, 16]. As a versatile mediator, Sema3A affects diverse signaling pathways such as MAPK, PI3K, STAT and small GTPases through a receptor complex containing neuropilin-1 (Nrp1) as its direct binding partner [17-19]. In the context of allergic diseases, expression of Sema3A was decreased in atopic dermatitis and allergic rhinitis specifically in the nasal epithelium of rhinitis mouse model. Interestingly, Sema3A treatment alleviated symptoms in both allergic disorders [20, 21]. However, the role of Sema3A-Nrp1 axis in ASM cell remodeling has not been investigated.

In this study we investigated the expression of Sema3A binding receptor Nrp1 on HASMC *in vitro* and *ex vivo*; and evaluated effect of Sema3A treatment on human ASM cell (HASMC) proliferation. We revealed that HASMC constitutively express Nrp1 *in vitro*. ASM bundle within bronchial biopsies from mild, moderate and severe asthmatics display Nrp1 expression. Sema3A significantly inhibited HASMC proliferation induced by platelet-derived growth factor (PDGF) via suppressing PDGFR, STAT3 and GSK-3β phosphorylation, and Rac1 GTPase activation. Our data suggest that Sema3A and its receptor are involved in the regulation of ASM hyperplasia in chronic airway diseases such as asthma.

## RESULTS

### Nrp1 is expressed by HASMC in healthy and asthmatic conditions

**Figure 1:**
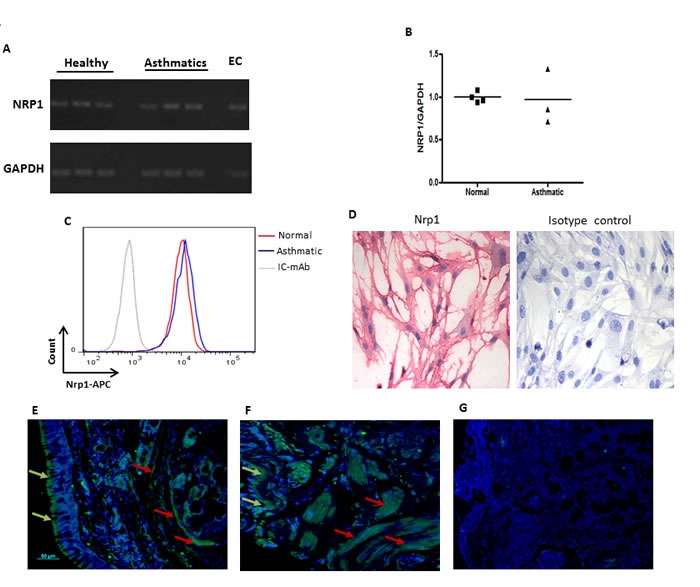
Expression of Nrp1 on HASMC *in vitro* and within bronchial biopsies Basal mRNA expression of NRP1 on primary HASMC was examined by RT-PCR using specific primers **A.** NRP1 mRNA level was also compared between healthy vs asthmatic HASMC by qPCR **B.** Surface expression of Nrp1 was compared between asthmatic and healthy bronchial HASMC by FACS analysis **C.** Immunocytochemistry was utilized to determine basal protein expression of Nrp1 in HASMC followed by visualizing slides under 100X magnification **D.** Staining with isotype control antibody showed no immunoreactivity in C and D. RNA and protein expression studies were performed on at least three different HASMC under the same conditions. Immunofluorescence staining was performed on paraffin-embedded bronchial biopsies obtained from healthy individuals **E.** and mild allergic asthmatics **F.** using rabbit anti-human Nrp1 mAb (*n* = 3 per group) followed by goat anti rabbit Alexa Fluor 488 and counterstaining with DAPI. No immunoreactivity was observed after staining with isotype antibodies **G.** Scale bar: 50 μm.

It has been previously shown that Sema3A binds directly Nrp1 expressed on the surface of target cells [17, 18]. Expression of Nrp1 on HASMC was evaluated at mRNA and protein levels. As shown in Figure 1A, mRNA for Nrp1 was expressed in primary HASMC from 3 different healthy and asthmatic donors. We further compared expression of Nrp1 between HASMC from healthy and asthmatic donors at both mRNA (Figure 1B) and protein (Figure 1C) levels. Nrp1 basal expression was also detected by immunocytochemistry in HASMC cells from healthy donors (Figure 1D) or asthmatics (data not shown). These data indicate that there is no significant difference in expression of Nrp1 between healthy donors and asthmatic HASMC at both mRNA and protein levels.

**Figure 2:**
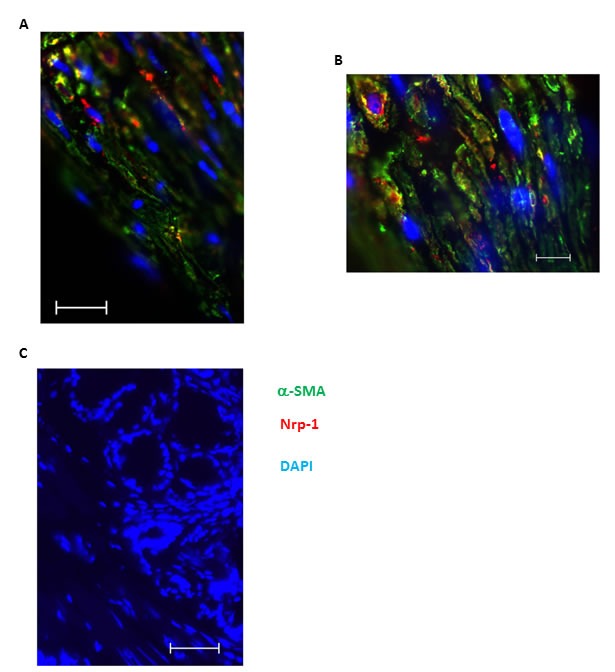
Nrp1 expression colocalizes with alpha smooth muscle positive cells in bronchial tissue of asthmatic individuals Double immunofluorescence staining was performed on paraffin-embedded bronchial biopsies obtained from severe allergic asthmatics to identify the expression Nrp1 using anti-human Nrp1 mAb. α-SMA was also stained as a specific smooth muscle marker to precisely demonstrate localization of Nrp1 on ASM bundles. **A.**-**B.** represent 400X and 1000X magnification of the same tissue, respectively. Scale bars in A-B are 20 and 10 μm, respectively. Counterstaining was performed with DAPI to visualise nuclear DNA in all slides and no immunoreactivity was observed after staining with isotype antibodies. Scale bar: 20 μm **C.**

To evaluate *in vivo* expression of Nrp1, we stained bronchial tissue sections obtained from healthy, mild and severe asthmatics using specific mAb. As shown in Figure 1E and 1F, Nrp1 is highly expressed in ASM bundles as well as surrounding airway epithelium of healthy and mild asthmatic patients, respectively. Similar results were obtained in severe asthmatics biopsies (data not shown). Tissue sections stained with isotype control antibody revealed no cross reactivity (Figure 1G). Furthermore, double immunofluorescence staining in lung tissue sections obtained from severe asthmatics showed that alpha smooth muscle actin (α-SMA) positively stained cells displayed Nrp1 immunoreactivity (Figure 2A-2B). Taken together, our data suggest that HASMC express Nrp1 both *in vitro* and *ex vivo*.

### Sema3A-Nrp1 axis abrogates PDGF-mediated HASMC proliferation

**Figure 3:**
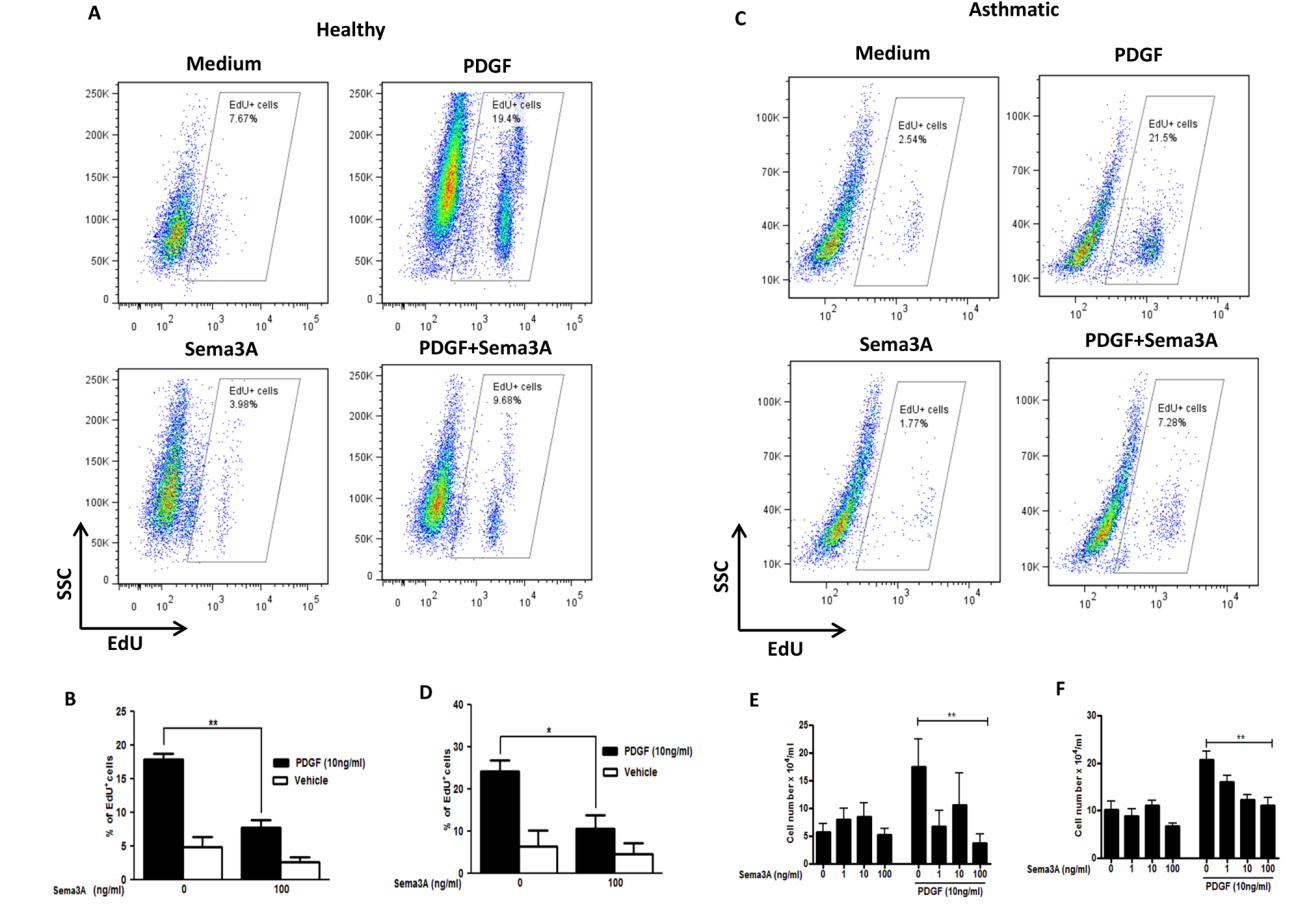
Inhibition of HASMC proliferation in response to Sema3A Basal and PDGF-mediated proliferation of primary healthy **A.** and asthmatic **C.** HASMC was studied by EdU incorporation assay 48 hours after stimulation. The results were quantified and the percentage of EdU^+^ cells representing DNA incorporation was statistically compared to corresponding control groups in healthy **B.** and asthmatic **D.** HASMC. Proliferation of healthy **E.** and asthmatic **F.** HASMC was studied by manual cell count before or 48h after PDGF±Sema3A stimulation. The results were quantified in a blind manner and statistically compared to control unstimulated or PDGF-stimulated groups; accordingly. EdU: 5-ethynyl-2′-deoxyuridine. The graphs are based on at least 3 independent experiments (*n* = 4 healthy and 3 asthmatic HASMC, *P < 0.05 **P < 0.01).

Sema3A is a potent anti-proliferative agent for Nrp1 expressing endothelial cells [22]. However, the effect of Sema3A on proliferation of ASM cells has not yet been investigated. To this aim, we treated serum-deprived HASMC with recombinant Sema3A with or without recombinant PDGF-BB (10 ng/ml). Then, incorporation of a fluorescent-labeled thymidine analogue, 5-ethynyl-2′-deoxyuridine (EdU), into newly synthesized DNA was evaluated. PDGF-induced proliferation was inhibited by Sema3A in HASMC from healthy (Figure 3A-3B) (*n* = 3, *P* < 0.01) and asthmatic donors (Figure 3C-3D) (*n* = 4, *P* < 0.05). As demonstrated in Figure 3B and 3D, Sema3A does not significantly affect basal proliferation of HASMC from neither healthy or asthmatic individuals; whereas it reduces PDGF-induced proliferation at 100 ng/ml (*n* = 4, *P* < 0.01). These results were further confirmed by performing manual cell count and trypan blue exclusion in HASMC isolated from healthy (Figure 3E) and asthmatic individuals (Figure 3F). In addition, we revealed that Sema3A inhibits proliferation of HASMC induced by not only PDGF but also epidermal growth factor (EGF) (data not sho wn).

**Figure 4:**
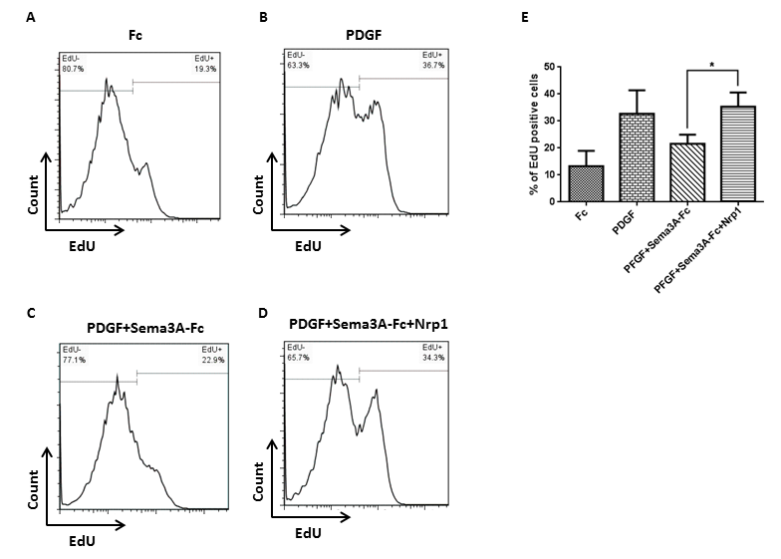
Nrp1-mediated fashion of Sema3A effect on HASMC proliferation HASMC were stimulated with Fc **A.** or PDGF **B.** as negative and positive control groups, respectively. HASMC were also treated with Sema3A-Fc and PDGF combination in the absence **C.** or presence **D.** of recombinant human Nrp1, followed by EdU incorporation assay. Sema3A inhibitory effect on PDGF-induced proliferation was significantly abrogated in presence of exogenous Nrp1 in three independent experiments **E.**

To determine whether Sema3A inhibitory effect on HASMC proliferation is mediated via Nrp1, cells were treated with recombinant Nrp1 and stimulated with PDGF or PDGF combined with Sema3A [13, 23]. Since recombinant human Sema3A was Fc conjugated, a control group treated with Fc alone was included; and this treatment did not affect HASMC proliferation (Figure 4). Treatment with Nrp1 significantly neutrralized anti-proliferative effect of Sema3A on HASMC at presence of PDGF (Figure 4) (*n* = 3, *P* < 0.05). Collectively, our studies suggest that Sema3A negatively regulates HASMC proliferation that involves Nrp1 receptor.

### Sema3A inhibits PDGFR tyrosine phosphporylation

**Figure 5:**
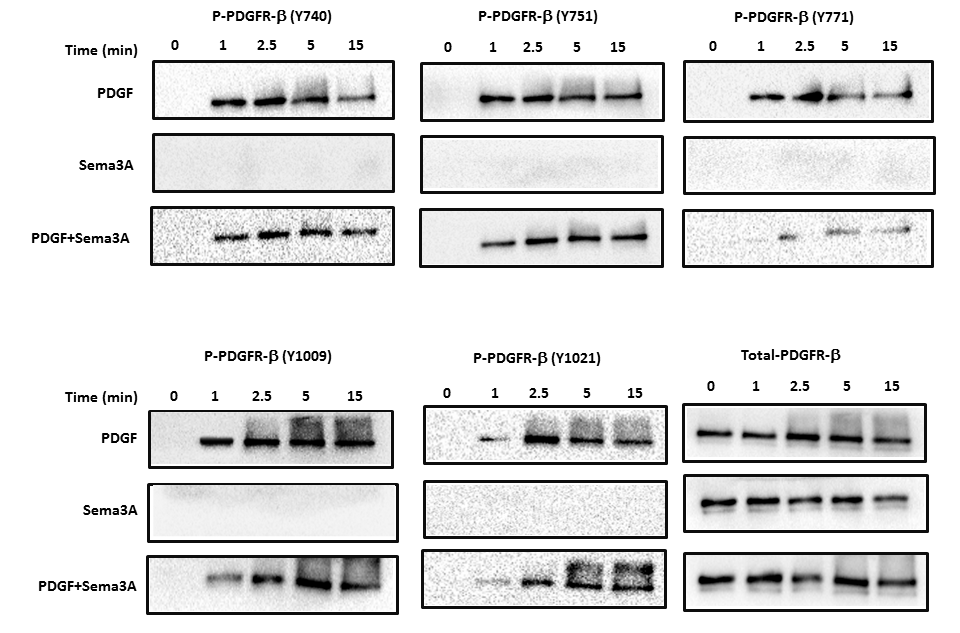
Sema3A inhibits PDGFR phosphorylation in HASMC Phosphorylation of PDGFR at 5 different sites was investigated in HASMC lysates upon Sema3A±PDGF stimulation at different time points by using specific antibodies. Total PDGFR was used as a loading control. PDGF stimulation robustly induced phosphorylation of its receptor while Sema3A alone did not affect PDGFR phosphorylation. Sema3A and PDGF co-stimulation reduced PDGF-induced phosphorylation of PDGFR at different time points.

Receptor tyrosine kinase, e.g. PDGFR β, phosphorylation plays a pivotal role in intracellular signal transduction activated upon growth factor stimulation [24]. Therefore, to understand the signaling mechanism by which Sema3A inhibits PDGF-BB mediated HASMC proliferation, we investigated whether Sema3A modulates phosphorylation of PDGF-BB specific receptor, PDGFR β. Our results revealed that Sema3A mediates its anti-proliferative effect partly through reduction of PDGFR β phosphorylation in different tyrosine residues (Figure 5). PDGF-BB-induced phosohorylation at Y771 was specifically reduced by Sema3A in all time points tested; while Y740, Y751, Y1009 and Y1021 phosphorylation was not affected by Sema3A treatment.

### Sema3A down-regulates PDGF-induced Rac1 GTPase activity

**Figure 6:**
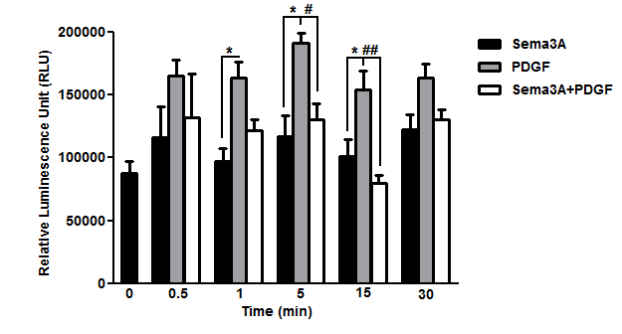
Suppression of PDGF-induced Rac1 GTPase activity by Sema3A in HASMC Rac1 GTPase activity was measured after Sema3A±PDGF stimulation by luminescent-based G-LISA at indicated time points. Sema3A suppressed PDGF-induced Rac1 GTPase activity 5 and 15 min after co-stimulation without significant effect on the basal activity. Data represent mean±SEM of five independent experiments performed at the same conditions. (*, # *P* < 0.05 and ## *P* < 0.01).

Rac1 GTPase has been previously shown as a key mediator which contributes to PDGF-induced ASM cell proliferation [5]. Therefore, Rac1 GTPase activity was quantified to elucidate the mechanism underlying HASMC proliferation upon Sema3A±PDGF stimulation by using G-LISA. As observed in Figure 6, PDGF increased Rac1 GTPase activity at indicated time points which was reduced to the basal level 5 and 15 min upon co-stimulation with Sema3A (*n* = 4, *P* < 0.01). Sema3A alone did not significantly affect HASMC proliferation which may explain its non-significant effect on basal HASMC proliferation in response to Sema3A.

### Sema3A inhibits GSK-3β and STAT3 phosphorylation in HASMC

**Figure 7:**
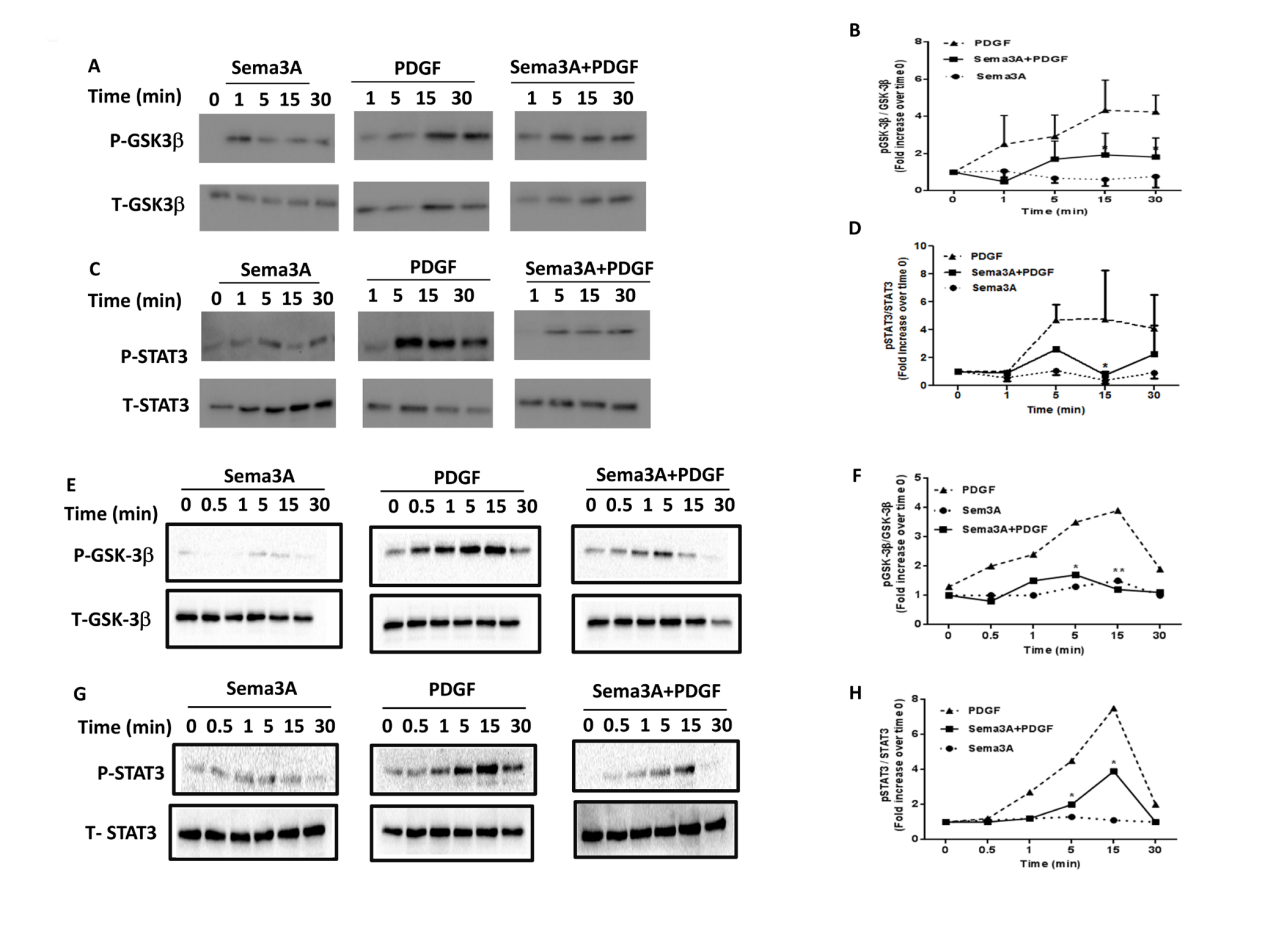
GSK-3β and STAT3 signaling pathways downstream of Sema3A anti-proliferative effect on HASMC isolated from healthy and asthmatics individuals Sema3A treatment induced a significant decrease in PDGF-induced GSK-3β **A.** and STAT3 **C.** phosphorylation in HASMC obtained from healthy individuals. Densitometric analysis was performed to quantify alterations in GSK-3β **B.** and STAT3 **D.** phosphorylation at indicated time points presented as the ratio of phospho over total compared with time zero. Data represent mean±SEM of three independent experiments on three different HASMC (*P < 0.05). Sema3A treatment induced a significant decrease in PDGF-induced GSK-3β (**E.**-**F.**) and STAT3 **G.**-**H.** phosphorylation in HASMC obtained from asthmatic patients. P: Phospho, T: Total, GSK-3β. Glycogen Synthase Kinase 3 beta, STAT3: Signal Transducer and Activator of Transcription 3.

As reported previously, PDGF induced phosphorylation of both GSK3-β [25] and STAT3 [26] in HASMC which lead to induction of proliferation. Sema3A treatment significantly decreased PDGF-mediated phosphorylation of GSK3-β and STAT3 (Figure 7C-7D) in HASMC isolated from healthy (Figure 7A-7D) and asthmatic (Figure 7E-7H) donors. Similar to Rac1 GTPase signaling, Sema3A alone did not influence GSK-3β and STAT3 signaling in HASMC (Figure 7). These data suggest that PDGFR, GSK3-β and STAT3 activation are negatively regulated by Sema3A treatment which inhibits PDGF-mediated HASMC proliferation in both healthy and asthmatic conditions.

## DISCUSSION

Dysregulated ASM cell proliferation leads to increased ASM mass which is associated with airway narrowing in some airway disorders including asthma, chronic obstructive pulmonary disease (COPD) and cystic fibrosis [2]. Deciphering molecular mechanisms underlying ASM cell proliferation may provide us with novel therapeutic clues. Semaphorins are versatile proteins involved in development of cancers, autoimmune and allergic disorders by affecting various cellular processes. Sema3A has been reported to be a potential candidate to treat experimental rheumatoid arthritis and different types of cancer through modulation of angiogenesis, cell proliferation, migration and adhesion [15, 16, 27-29]. However, its potential contribution to the regulation of airway remodeling has not been investigated.

Here, we revealed that the Sema3A receptor, Nrp1, is expressed on HASMC from both healthy and asthmatic subjects suggestive of a functional contribution in ASM biology. We further confirmed expression of Nrp1 on human ASM bundles in healthy and asthmatic lung tissue sections indicating a potential unknown role of Sema3A-Nrp1 axis *in vivo*. However, comparison between healthy and asthmatic HASMC revealed similar levels of Nrp1 mRNA and protein surface expression. Expression of Nrp1 has been extensively studied in visceral smooth muscle cells but not in those of ASM cells [30-32]. Selective deletion of Nrp1 encoding gene in mouse visceral smooth muscle cells leads to gastrointestinal contractility and motility wherein a less contractile phenotype in aged mice have been reported [32]. Our functional studies demonstrated that exogenous recombinant Sema3A inhibits PDGF-induced HASMC proliferation. We observed that Sema3A exerts the anti-proliferative effect on both tracheal and bronchial HASMC proliferation. Treatment with Sema3A alone did not significantly inhibit HASMC proliferation at the baseline.

Blockade of Sema3A inhibitory effect by exogenous human recombinant Nrp1 treatment suggests that Sema3A signaling might be initiated through this receptor on HASMC surface. It should be mentioned that the repulsive role of Sema3A in growth cone guidance during development of nervous system is mediated through Nrp1 and plexinA1 as the ligand-binding and signal transducing elements, respectively [33]. In fact, according to preceding studies, short intracellular domain of Nrp1 necessitates a requirement for plexin co-receptors to convey Sema3A signal [33, 34]. From a mechanistic point of view, Sema3A inhibitory effect on HASMC proliferation was mediated via targeting intracellular signaling pathways including PDGFR tyrosine phosphprylation, Rac1 GTPase, GSK-3β and STAT3.

Interaction of PDGF-BB with the PDGFR induces dimerization which is essential for the activation of tyrosine kinase receptors [35]. Subsequently, PDGFR undergoes auto-phosphorylation in specific tyrosine residues as an early signaling event which activates intracellular signal transduction upstream of multiple pathways such as Ras/Rac small GTPases, MAPK, PI3K, STAT and Src signaling thereby modifying cellular functions such as cell proliferation and migration [36, 37]. For instance, it has been shown that differential phosphorylation pattern of tyrosine residues on PDGFR upon PDGF stimulation induces switching from proliferation to migratory signaling in glioblastoma cell [38] and for both functions in human aortic smooth muscle cells [39]. In our study, we specifically found that PDGFR phosphorylation at Y771 induced by the ligand is decreased upon sema3A treatment. Previous studies have demonstrated that Y771, but not other subunits, is required for efficient binding to GTPase activating protein (GAP) [40, 41] suggesting an important role of this phosphorylation in downstream events such as Rac1 GTPase signaling.

Previous studies have demonstrated the non-redundant role of GSK-3β pathway in HASMC proliferation [25]. In addition, Sema3A repulsive effect on axon guidance and neuronal polarization has been shown to be associated with abrogation of GSK-3β signaling in a manner dependent on the phosphatase activity of a tumor suppressor PTEN (phosphatase and tensin homologue deleted on chromosome 10) [42, 43]. However, to our knowledge, the inhibitory effect of Sema3A on GSK-3β signaling in ASM cells has not been addressed, so far.

STAT3 is also a key signaling element of HASMC proliferation which has been shown to physically interact with Rac1 GTPase in these cells [5]. As a novel finding, we revealed for the first time that Sema3A may negatively regulate PDGF-induced tyrosine phosphorylation of STAT3. Small GTPase Rac1 is a key mediator of ASM cell proliferation which is regulated by Sema3A in neurons [44] and also in platelets [45]. However, the involvement of Rac1 as a part of signaling mechanism underlying anti-proliferative effect of Sema3A on HASMC had not been addressed previously. The short intracellular domain of Nrp1 may partly explain the different time points of suppression of Rac1 GTPase activity between Sema3E [13] and Sema3A on HASMC. In our previous study, direct interaction of Sema3E with plexinD1 reduced Rac1 GTPase activation 1 min after co-stimulation with PDGF which occurred 5 min after Sema3A and PDGF co-stimulation of HASMC at the same conditions [13].

It has been demonstrated that Sema3A inhibits dendritic cell (DC)-induced T cell proliferation via affecting polymerization of cytoskeletal filamentous (F)-actin and early events of T cell activation [46]. In fact, Sema3A blocks polarization of T cell receptor (TCR) as well as phosphorylation of zeta-chain-associated protein kinase 70 (ZAP-70) and focal adhesion kinase (FAK) [46]. It suggests that Sema3A could be considered to modulate, not only ASM hyperplasia but also, airway inflammation in allergic asthma. In addition, Sema3A has also emerged as an essential player in bone remodeling in which it inhibits osteoclast differentiation through suppression of phospholipase C gamma (PLCγ) activation and calcium oscillation. In that study, Sema3A activates Rac1 and canonical Wnt signaling in osteoblast differentiation suggestive of a context-dependent manner of Sema3A-Nrp1 signaling [47]. Another important issue relevant to this study is the ability of Nrp1 extracellular domain to bind several growth factors such as transforming growth factor β1 (TGF-β1) [48], hepatocyte growth factor (HGF) (20), some isoforms of the fibroblast growth factor (FGF) [49], and PDGF [50, 51]. Therefore, it can be speculated that besides direct inhibitory effects of Sema3A-Nrp1 interaction on PDGF-induced HASMC proliferation, Nrp1 might compete with PDGFR to bind its ligand which, unlike cancer studies [52], further weakened the cellular function. In fact, the repulsive role of Nrp1 ligation by Sema3A in HASMC is more similar to its function in the immune system, e. g. plasmocytoid DC (pDC) [53] and regulatory T cells [54], in which it exerts immunomodulatory effects [55].

The finding that the Sema3A-Nrp1 inhibitory axis is an upstream effector of the signaling cascade Rac1, GSK-3β and STAT3 provides an additional regulator of HASMC proliferation which had not been shown previously. In this study, we established Sema3A as a novel regulator of HASMC proliferation by suppressing Rac1, GSK-3β and STAT3 signaling through direct binding to Nrp1. However, other signaling pathways involved in regulation of HASMC proliferation such as PDGFR, MAPK/ERK and PI3K/Akt may also be negatively affected upon Sema3A treatment. Future studies may elucidate the potential role of other intracellular signaling components in Sema3A-inhibited HASMC proliferation.

Indeed, Sema3A and signaling mediators studied here are ubiquitously expressed in several cell types beyond HASMC and play diverse roles at both homeostatic and pathological conditions in other tissues. Therefore, specific targeting of HASMC should be carefully considered when designing novel *in vivo* strategies to treat ASM hyperplasia based on Sema3A-Nrp1 axis and its downstream signaling. In summary, our study established Sema3A as a novel regulator of HASMC proliferation by suppressing Rac1, GSK-3β and STAT3 signaling mediated by Nrp1. Our findings raise the possibility of using Sema3A as a new therapeutic target to inhibit HASMC proliferation leading to minimize airway remodeling.

## MATERIALS AND METHODS

### Human subjects

Bronchoscopy was performed according to procedures approved by the Human Research Ethics Board of Laval University, Quebec, Canada. Written informed consent was obtained from each individual. Bronchial biopsies were obtained from healthy, mild/moderate, and severe asthmatic individuals fulfilling the American Thoracic Society selection criteria. The clinical characteristics are depicted in Table 1.

**Table 1:** Clinical characteristics of individuals subjected to bronchoscopy

Severity	Gender	Age	Smoking status	Allergy	Medication	Dose (μg/day)	PC20 (mg/ml)	FEV1 (%)
Normal #1	M	19	No	No	No		>128	112
Normal #2	F	20	No	No	No		>128	102
Mild #1	F	21	No	Yes	Salbutamol or Terbutaline	prn	0.68	84
Mild #2	F	20	No	Yes	Salbutamol or Terbutaline	prn	0.3	99
Mild #3	M	18	No	Yes	Salbutamol or Terbutaline	prn	0.66	76
Severe #1	F	21	Ex	Yes	Terbutaline Budesonide	prn 1200	0.16	103
Severe #2	M	37	No	Yes	Fluticasone-Salmetrol (Advair 250) Beclomethasone Montelukast	1000 1200 10	ND	81

PC20: Provocative concentration of methacholine causing a 20% fall in FEV1

FEV1: Forced Expiratory Volume in 1 second

Ex: Ex-smoker, prn: per need

Primary HASMC were obtained from healthy non-asthmatic and asthmatic individuals in accordance with procedures approved by the Human Research Ethics Board of the University of Manitoba, Winnipeg, Canada and the Ethics Committee at University Hospital Basel, Switzerland. The clinical characteristics of asthmatic patients used to isolate HASMC have been mentioned in Table 2.

**Table 2:** Clinical profile of asthmatic patients used for bronchial HASMC isolation

Patient	Gender	Age	FEV1%	Medication	MEF25	MEF50	MEF75
Subject #1	M	30	81	Seretide	39	59	59
Subject #2	M	42	80	Symbicort	28	43	85
Subject #3	M	42	78	Symbicort	25	48	79

FEV1: Forced Expiratory Volume in 1 second

MEF: Maximal Expiratory Flow

### Reagents

Recombinant human Sema3A, Nrp1 and PDGF-BB as well as APC-conjugated mouse anti-human Nrp1 monoclonal antibody and also sheep anti-human Nrp1 affinity purified polyclonal antibody were purchased from R&D Systems (Minneapolis, MN) and FITC-conjugated anti-human Nrp1 was from Abcam (Toronto, ON, Canada). Cell culture media were obtained from Invitrogen (Burlington, Ontario, Canada). FBS and Insulin-Transferrin-Selenium-X (ITS-X) were purchased from HyClone Laboratories (Logan, UT) and Invitrogen, respectively. All other reagents are from Sigma-Aldrich unless otherwise indicated.

### HASMC culture

HASMC were isolated from lower tracheal section of healthy non asthmatic donors from whom recipient cannot be matched. Asthmatic bronchial HASMC were isolated from dissected ASM bundles of the bronchial biopsies from mild asthmatics. The patients did not manifest exacerbations and received no medication 1 week before bronchoscopy. HASMC were used at passage 2-5 in all experiments. HASMC were grown on plastic dishes in DMEM containing 10% FBS to reach about 70% confluency and then serum starved to synchronize for 48 h in Ham's F12 supplemented with ITS.

### RT-PCR and quantitative real-time PCR

Total RNA was extracted from HASMC using TRIzol™ (Life Technologies). 2 μg of RNA was subjected to MultiScribe™ Reverse Transcriptase to synthesize cDNA. Expression of NRP1 and GAPDH was analyzed by conventional RT-PCR in healthy primary HASMC. Expression of NRP1 was also compared between healthy and asthmatic HASMC by using qPCR. GAPDH was used to normalize mRNA quantification. Expression values and statistical significance were calculated using the 2^-ΔΔCt^ method. All RT- and q- PCR experiments were performed using the following primers. The forward and reverse-specific primer sequences and the size of the amplicon for NRP1 were FWD5'TATTCCCAGAACTCTGCCC3'and REV5'TGTCATCCACAGCAATCCCA3’, 253 bp and for GAPDH were FWD5'AGCAATGCCTCCTGCACCACCAAC3'and REV 5’-CCGGAGGGGCCATCCACAGTCT-3’, 137 bp. The annealing temperature and the number of cycles for both NRP1 and GAPDH were 60°C and 30 cycles.

### Flow cytometric analysis of Nrp1 surface expression

Primary cultured HASMC were detached using Versene^®^ (0.02% EDTA, Lonza, Walkersville, MD). 1x10^5^ HASMC were incubated with either APC-conjugated mouse anti-human Nrp1 or IgG2a as isotype control for 30 min at 4°C. The cells were washed twice, resuspended in flow buffer and acquired on FACSCanto™ II flow cytometer (BD Biosciences, San Jose, CA). Nrp1 surface expression was analyzed by FlowJo software (Tree Star, Ashland, OR).

### Immunocytochemistry

HASMC were seeded onto sterile glass coverslips, fixed in 4% paraformaldehyde; permeabilized and then non-specific Ab binding was blocked. Next, cells were labelled with sheep anti-human Nrp1 Ab or either sheep IgG and incubated overnight at 4°C. HASMC were incubated with biotin labelled rabbit anti-sheep IgG followed by addition of streptavidin-alkaline phosphatase for 1 hour. Development and counterstaining were performed by using Fast-red and modified Mayer's haematoxylin (Fisher Scientific, Fair Lawn, NJ), respectively. Finally, mounted coverslips were visualized by AxioVision software (Carl Zeiss, Inc.).

### Immunofluorescence

Paraffin embedded sections were dewaxed in xylene and then rehydrated through graded concentrations of alcohol to water. Antigen retrieval was performed by boiling for 10 min in sodium citrate buffer (pH, 6.0). Sections were incubated with blocking buffer (1% BSA, gelatine 1% human healthy serum in TBS) for 1 h at room temperature. After 3 washes with TBS, sections were incubated with rabbit anti-human Nrp1 (10 μg/ml) at 4°C overnight. For double immunofluorescence staining, sections were incubated with both rabbit anti-human Nrp1 and mouse anti-human alpha smooth muscle actin (αSMA) (4 μg/ml) Ab. After washing, sections were incubated with either goat anti-rabbit Alexa 488 alone or with goat anti-mouse Alexa 568 for 1 hr at room temperature, followed by washing and mounting with an anti-fade containing 4′,6-diamidino-2-phenylindole (DAPI). Sections were visualized with Zeiss microscope by using AxioVision software (Carl Zeiss, Inc, Thornwood, NY).

### EdU cell proliferation assay and cell count

Serum-starved HASMC were stimulated with Sema3A at presence or absence of PDGF and cell proliferation was assessed using Click-iT™ EdU flow cytometry assay kit (Life Technologies). Briefly, 10μM of a thymidine analogue 5-ethynyl-2′-deoxyuridine reagent was added to HASMC 16 h after stimulation and cells were harvested 24 h later. Cells were fixed, permeabilized and then stained with Click-iT reaction cocktail containing copper sulphate and fluorescent dye azide (Alexa Fluor^®^ 488). Finally, EdU incorporation into newly synthesized DNA as an indicator of cell proliferation was measured by flow cytometry. To further confirm EdU incorporation data, serum-starved HASMC were treated with different concentrations of Sema3A (0, 1, 10 and 100 ng/ml) with or without PDGF (10 ng/ml) in a different set of experiments. In some EdU incorporation experiments, a control group was included in which HASMC were stimulated with the same Fc subclass as Sema3A-Fc to demonstrate that Fc portion does not influence HASMC proliferation. After 48 h, HASMC were counted manually using a hemocytometer and cell viability was determined by trypan blue exclusion in a randomized manner.

### Measurement of Rac1 GTPase activity

Rac1 GTPase activity was assessed in HASMC upon Sema3A±PDGF stimulation using a G-LISA according to manufacturer's instructions (Cytoskeleton, Inc. Denver, CO). Snap-frozen HASMC lysates in liquid nitrogen were subjected to Rho binding domain of Rac1 which was pre-coated in a Rac-GTP affinity 96-well plate. Then, Rac1-GTP as the active form was detected using primary and HRP-conjugated secondary Abs followed by chemiluminescent development.

### Western blot analysis

Protein lysates obtained from G-LISA were used for PDGF receptor (PDGFR), GSK-3β and STAT3 signaling studies. 10 μg of each lysate was loaded on 10% SDS-PAGE and transferred to PVDF membranes. Non-specific binding sites were blocked by adding non-fat milk followed by overnight incubation with phosphorylated GSK-3β (S9) and STAT3 (Y705) at 4°C. PDGFR β phosphorylation was studied by using an antibody sampler kit (Cell Signaling Technology, Boston, MA). It contains specific monoclonal antibodies against total form of PDGFR β as well as five tyrosine phosphorylation sites (Y740, Y751, Y771, Y1009 and Y1021) on PDGFR β. Then, blots were incubated with HRP-conjugated secondary Abs and bands were visualized with ECL reagents. Total anti-PDGFR β, GSK-3β and STAT3 were used as loading controls. Densitometric analysis was performed by using Image Lab™ 4.1 software (Bio-Rad Laboratories Ltd., Mississauga, ON, Canada) and integrated density value was presented as the fold-increase in phosphorylated over total forms of GSK-3β and STAT3 compared to time zero.

### Statistical analysis

GraphPad Prism 5.0 software was used for statistical analysis and values were presented as the mean±SEM of at least three independent experiments. Data were analyzed by *t* test, one-way or two-way ANOVA. Bonferroni's multiple comparison post-hoc analysis was also performed. Differences were considered to be statistically significant at ^#^ and * *P* < 0.05, ^##^ and ** *P* < 0.01.

## Abbreviations

ASM: Airway smooth muscle
EdU: 5-ethynyl-2′-deoxyuridine
GTPase: Guanosine triphosphatase
GSK-3β: Glycogen synthase kinase-3 beta
HASMC: Human airway smooth muscle cells
Nrp1: Neuropilin 1
PDGF: Platelet-derived growth factor
PDGFR: Platelet-derived growth factor receptor
Rac1: Rho-related C3 botulinum toxin substrate 1
Sema3A: Semaphorin 3A
STAT3: Signal Transducer and Activator of Transcription 3
